# Lipid and Glycemic Profiles in Patients with Bipolar Disorder: Cholesterol Levels Are Reduced in Mania

**DOI:** 10.3390/medicina57010028

**Published:** 2020-12-30

**Authors:** Laura Fusar-Poli, Andrea Amerio, Patriciu Cimpoesu, Antimo Natale, Virginio Salvi, Guendalina Zappa, Gianluca Serafini, Mario Amore, Eugenio Aguglia, Andrea Aguglia

**Affiliations:** 1 Department of Clinical and Experimental Medicine, Psychiatry Unit, University of Catania, 95123 Catania, Italy; antimo.natale88@gmail.com (A.N.); eugenio.aguglia@unict.it (E.A.); 2 Department of Neuroscience, Rehabilitation, Ophthalmology, Genetics, Maternal and Child Health, Section of Psychiatry, University of Genoa, 16123 Genoa, Italy; andrea.amerio@unige.it (A.A.); patriciu.cimpoesu@gmail.com (P.C.); guendalina.zap@gmail.com (G.Z.); gianluca.serafini@unige.it (G.S.); mario.amore@unige.it (M.A.); andrea.aguglia@unige.it (A.A.); 3 IRCCS Ospedale Policlinico San Martino, 16123 Genoa, Italy; 4 Department of Psychiatry, Tufts University, Boston, MA 02111, USA; 5 Department of Clinical Neurosciences, Polytechnic University of Marche, 60121 Ancona, Italy; virginiosalvi@gmail.com

**Keywords:** cholesterol, triglycerides, metabolic syndrome, glucose, mania, bipolar disorder

## Abstract

*Background and Objectives*: Bipolar disorder (BD) is a severe mental condition with a lifetime prevalence estimated around 2% among the general population. Due to risk factors, etiological mechanisms, and the chronic use of psychotropic medications, people with BD are frequently affected by medical comorbidities, such as metabolic syndrome (MetS), associated with altered blood levels of glucose, cholesterol, and triglycerides. Moreover, the lipid concentration may be associated with the severity of psychiatric symptoms. *Materials and Methods*: Five hundred and forty-two in- and outpatients (418 affected by BD and 124 affected by schizophrenia) were recruited in two Italian university hospitals. A blood examination assessing the fasting glucose, total cholesterol, high-density lipoprotein (HDL) cholesterol, low-density lipoprotein (LDL) cholesterol, and triglycerides was performed. *Results*: No significant differences were found in the lipid and glycemic profiles between patients with BD and schizophrenia. When considering only the BD sample, we found that patients experiencing a manic episode had significantly lower total cholesterol, HDL, and LDL than euthymic patients. Moreover, the total and LDL cholesterol levels were significantly lower in (hypo)manic than depressed patients. Mood episodes did not influence the triglyceride and glucose levels in our sample. *Conclusions*: Clinicians should pay attention to blood cholesterol levels in patients with BD, as differences in concentrations may predispose them to severe medical conditions and can be associated with the onset of mood episodes.

## 1. Introduction

Bipolar disorder (BD) is a group of affective disorders characterized by the presence of recurring manic or hypomanic episodes that may alternate with major depressive episodes [1]. Recent epidemiological studies reported that the lifetime prevalence of BD can be estimated around 2.4% [2]. The Diagnostic and Statistical Manual of Mental Disorders, Fifth Edition (DSM-5) includes the category “Bipolar and related disorders”, which encompasses BD type I, BD type II, and cyclothymic disorder. Atypical bipolar-like phenomena that do not fit the canonical subtypes are included in the “other specified and bipolar-related disorders” category [3].

Given its features and prevalence, BD ranks as the 17th leading source of disability among all diseases worldwide [4]. Moreover, it is associated with high rates of premature mortality due to both medical comorbidities and high suicide rates [1]. Millions of people affected by BD adhere to psychopharmacological treatments during the lifespan. Medications such as mood stabilizers, oral and long-acting antipsychotics, and antidepressants are recommended by international guidelines and used on- and off-label both for the treatment of acute mood episodes and the prevention of relapses [5,6]. However, psychotropic medications are not free from side effects, which may include hepatic malfunctions, renal, thyroid and parathyroid dysfunctions, and metabolic syndrome (MetS) [1,7,8,9,10]. 

MetS is a clustering of at least three of the following five medical conditions: abdominal obesity, high blood pressure, high blood glucose, high serum triglycerides, and low serum high-density lipoprotein (HDL). The prevalence of MetS increases the risk of developing both cardiovascular disease and type 2 diabetes [11,12]. A meta-analysis by Vancampfort et al. reported that the overall MetS prevalence in patients with BD was 37.3% [13]. Moreover, 13.7% of patients with BD were reported to suffer from diabetes mellitus, indicating high glucose blood levels [14]. Indeed, BD and MetS share many features, including endocrine disturbances, sympathetic nervous system dysregulation, and behavioral patterns, such as physical inactivity and overeating [15]. Additionally, as mentioned before, the use of psychotropic medications may cause or worsen MetS. In fact, it has been shown that the rate of MetS is significantly higher in patients treated with antipsychotics (45.3%) than in patients who are antipsychotic-free (32.4%) [13], even if an increased risk for MetS has been also reported in drug-naïve patients [16].

Importantly, the presence of MetS—and, thus, the level of lipids and glucose—seems to be related not only to the general medical status but, also, to the psychiatric status of people with BD. For instance, it has been reported that patients with comorbid BD and MetS undergo more hospitalizations and show poorer insight and global functioning [17]. Other authors have argued that lipid profiles, in particular, are linked to suicide attempts and their lethality in patients with severe mental disorders, including BD. However, evidence is still contrasting [18,19,20,21].

Many authors investigated the lipid and glycemic profiles in patients with mood alterations, particularly major depressive disorder (MDD) [22,23,24,25]. A recent meta-analysis, including 11 case control studies, showed that first-episode MDD was associated with significantly increased triglyceride levels and lower HDL cholesterol levels, while no significant alterations in low-density lipoprotein (LDL) and the total cholesterol levels were found [26]. Nevertheless, only few researchers specifically compared these parameters between BD and other severe mental disorders or between different BD phases (i.e., mania, depression, and euthymia).

Interestingly, Wysokiński et al. [27] considered both the primary diagnosis (schizophrenia, BD, and depression) and the current mood episode (unipolar depression, bipolar depression, or mania). The authors found that triglycerides levels were significantly higher in bipolar depression than schizophrenia, while they were reduced in mania. Additionally, the total cholesterol levels seemed increased in patients with BD compared to psychotic patients, while LDL cholesterol was significantly lower in manic patients compared to both bipolar depression and schizophrenic patients. In 2007, Chung et al. [28] measured the serum cholesterol and triglyceride levels in patients with BD type I hospitalized for acute mood episodes (68 manic, eight depressive, and six mixed). The authors found that severe depressive symptoms were associated with higher serum cholesterol levels. Conversely, a negative association was noted between the serum triglyceride levels and overall psychiatric symptoms [28]. More recently, Huang et al. [29] examined the lipids and glucose level of 32 manic BD patients, 32 depressed BD patients, and 64 healthy controls. The authors found that the mean cholesterol level in manic participants was significantly lower than in depressed participants. Additionally, the lowest rate of dyslipidemia (hypertriglyceridemia or LDL cholesterol) was observed in acute bipolar mania [29]. To our knowledge, only a few authors have explored the differences in glucose and lipid levels between patients with BD and other severe mental illnesses. Moreover, the lipid and glycemic profiles during different mood episodes in BD have been barely studied. Thus, the aims of the present paper were:

(1) To compare the lipid (i.e., total, LDL, HDL cholesterol, and triglycerides) and glycemic profiles (i.e., glucose) between patients affected by BD and a control sample of patients affected by schizophrenia.
(2) To evaluate the differences in the lipid and glycemic profiles during the different mood episodes in patients affected by BD (i.e., depression, mania, and euthymia).

## 2. Materials and Methods

We conducted a cross-sectional study in two Italian university psychiatry units (Genoa and Catania). The sample included 542 subjects with a primary diagnosis of BD or schizophrenia referred to in- and outpatient units between January 2017 and October 2020.

### 2.1. Participants

We included subjects aged > 18 years and a primary diagnosis of BD or schizophrenia according to the DSM-5 [3]. The euthymic phase of BD was defined as a Young Mania Rating Scale (YMRS) score ≤ 12 [30] and a Montgomery-Åsberg Depression Rating Scale (MADRS) ≤ 10 [31] with psychopathological conditions stable for at least six months.

All subjects were followed in the respective in- and outpatient services. Participants expressed a willingness to take part in the study by signing a written consent after the aims of the study and study procedures were thoroughly explained. All subjects were Caucasian of Italian origin. 

Exclusion criteria were: (1) any condition affecting the ability to fill out the assessment, such as major neurocognitive disorders, (2) any severe neurological disorders, including an intellectual disability, (3) refusal of written informed consent, (4) a history of active substances abuse/dependence during the past 6 months, (5) pregnancy or recently given birth, and (6) acute and severe medical comorbidity.

The study design was conducted in accordance with the guidelines provided in the current version of the Declaration of Helsinki. The protocol was regularly approved by the Ethical Committee of IRCCS San Martino, Genoa, with number 82/13, amended on 10 February 2017, and registered with as number 028 on 2 March 2017.

### 2.2. Assessment and Procedures

A form including sociodemographic and clinical characteristics such as gender; current age; marital and occupational status; educational level; primary diagnosis (BD or schizophrenia); bipolar illness phase (manic, depressive, or euthymic); and current pharmacological treatment was completed by participants’ psychiatrists. Therefore, a blood drawing for a routine blood exam was performed at hospital admission for inpatients, while, for outpatients, the results of previous blood examinations were considered valid if the last blood sample was drawn within 2 months before entry in the study. At the time of blood drawing, patients fasted for the previous 10 h. A blood examination assessing the fasting glucose, total cholesterol, HDL and LDL cholesterol, and triglycerides was performed.

### 2.3. Statistical Analysis

All statistical analyses were performed using SPSS version 25.0 (IBM Corp., Armonk, NY, USA), and the value of statistical significance was set at *p* < 0.05 (two-tailed).

Continuous variables were represented as means and standard deviation (SD), while categorical variables were represented as frequency and percentage considering the sociodemographic and clinical characteristics.

Firstly, the sample was divided into two subgroups: the first consisted of subjects with a primary diagnosis of BD, while the second subgroup was characterized by subjects with a primary diagnosis of schizophrenia. For the comparison, the Student’s *t*-test for independent samples was used for continuous variables and Pearson’s chi-square test (χ^2^) for categorical variables.

Subsequently, we considered only the subjects with a primary diagnosis of BD, and the sample was divided according to the illness phase into three subgroups: (a) (hypo)manic episode, (b) major depressive episode, or (c) euthymic phase. In order to analyze the differences between these three subgroups, we used ANOVA with Tukey’s post-hoc correction for continuous variables and Pearson’s chi-square test (χ^2^) for categorical variables, respectively. 

## 3. Results

### 3.1. General Characteristics of the Sample

The total sample comprised 542 participants, of which 77.1% had a primary diagnosis of BD (N = 418). The mean (±SD) age of the total sample was 52.62 (±12.87) years, and 266 patients (49.1%) were male. Each participant was taking, on average, 3.63 (±1.18) medications at the assessment. 88.7% were treated with at least one antipsychotic, 36.1% were receiving at least one antidepressant, and 37.4% and 31.1% were treated with valproate and lithium, respectively. All sociodemographic and clinical characteristics, including the metabolic parameters of the overall sample, are displayed in Table 1.

### 3.2. Comparison of Sociodemographic, Metabolic, and Pharmacological Parameters between Patients with BD and Schizophrenia

When comparing the metabolic parameters according to the primary diagnosis, no statistical differences were found. Regarding pharmacological treatment, bipolar subjects were receiving a significantly higher number of psychiatric medications (3.80 ± 1.18 vs. 3.11 ± 0.98, *p* < 0.001)—specifically, all investigated mood stabilizers, antidepressants (42.7% vs. 16.5%, *p* < 0.001), and benzodiazepines (66.5% vs. 43.0%, *p* < 0.001). Schizophrenic patients were taking more antipsychotics (99.2% vs. 85.2%, *p* < 0.001)—in particular, typical (39.7% vs. 17.3%, *p* < 0.001) and long-acting antipsychotics (32.2% vs. 6.4%, *p* < 0.001). No difference in terms of atypical antipsychotic treatment was detected. All findings are reported in Table 2. 

### 3.3. Serum Levels of Glucose, Cholesterol, and Triglycerides in Different Phases of Bipolar Disorder

Considering only subjects with a primary diagnosis of BD, and after excluding the patients for whom no information regarding the current mood episode was available (N = 171), we investigated the differences in the levels of glucose, cholesterol, and triglycerides among patients experiencing different BD phases. The results are depicted in Table 3 and show that the levels of total, HDL, and LDL cholesterol were significantly higher in the (hypo)manic than the euthymic phase. Conversely, patients experiencing a major depressive episode had significantly higher levels of total and HDL cholesterol compared to (hypo)manic patients. No significant differences were detected between participants with bipolar depression and the euthymic subjects.

## 4. Discussion

### 4.1. Main Findings

Psychiatric disorders are frequently burdened by a number of medical comorbidities and metabolic abnormalities that may worsen their quality of life and cause a reduction of life expectancy [9,13,17,32,33,34].

The present study primarily aimed to compare some components of MetS (i.e., total cholesterol, HDL, LDL, triglycerides, and glucose) in patients with BD and schizophrenia, two severe mental illnesses that share many genetic and environmental risk factors [35]. No significant differences were found in any of the metabolic parameters considered. So far, the findings of the scientific literature have been contrasting. Some authors reported an increased metabolic risk in patients with BD compared to individuals with schizophrenia [27,36,37]. Other authors argued no differences between these two groups [38]. Nevertheless, given the heterogeneous methodological procedures adopted in previous studies, it is difficult to critically compare our findings to the published literature.

It is established however that lipid and glycemic profiles are frequently altered in patients affected by both BD and schizophrenia [32]. The causes for the increased incidences of metabolic disorders are not completely understood. It has been suggested that underlying genetic mechanisms combined with a variety of environmental factors, including tobacco smoking, low physical activity levels, inadequate somatic healthcare services, and antipsychotic medications, may contribute to an increased metabolic risk [35]. Interestingly, So et al. recently proposed that metabolic abnormalities could be genetically associated with schizophrenia and independent from medication side effects. Conversely, cardiometabolic abnormalities in BD are more likely to be secondary to antipsychotic medication [35]. In fact, the BD patients included in our sample were taking, on average, a higher number of medications—particularly, antidepressants, mood stabilizers, and benzodiazepines. Even if typical and long-acting antipsychotics were mainly prescribed to psychotic patients, the proportion of BD subjects on antipsychotic treatment was averagely higher. Moreover, schizophrenic patients are frequently treated in monotherapy, while combined therapy is preferred in BD due to the “swinging” nature of the disease. Additionally, an association between antidepressants use (i.e., selective serotonin reuptake inhibitors, SSRI) and metabolic abnormalities in patients with schizophrenia or BD has been also reported [39,40,41]. 

Psychiatrists should dedicate more time to the evaluation and management of serum levels of lipids and glucose in people with BD. MetS is strictly related to incidences of cardiovascular and metabolic diseases, as well as to a reduction of life expectancy, in psychiatric patients. Importantly, an elevated prevalence of MetS and glucose intolerance has been also reported in patients newly diagnosed with BD [42]. Therefore, these conditions should be screened since the early stages to employ prompt and adequate care strategies that can reduce the risk of cardiometabolic complications and premature death, including complementary interventions acting on the lipid profile [43,44]. 

The second aim of the present study was to investigate whether being in a different phase of BD might influence the blood levels of triglycerides, cholesterol, and glucose. The findings are interesting, as the post-hoc comparisons showed lower total, LDL, and HDL cholesterol levels in (hypo)manic patients as compared to euthymic bipolar individuals; additionally, the total and LDL cholesterol levels were significantly lower during (hypo)mania than depression. By simplifying, we found an indirect correlation between mood and cholesterol level: the higher the mood, the lower the cholesterol, regardless of its lipoprotein constitution. Unfortunately, this finding was not supported by a quantification of the psychiatric symptom severities through the systematic administration of scales. Moreover, a link of causality between the mood episodes and cholesterol levels in BD could not be established due to the cross-sectional design of our study.

Our results are in-line with several reports that identified lower serum cholesterol levels in manic patients than healthy controls [45,46,47] or individuals with bipolar depression [27,28,29,48,49]. The underlying mechanism for the altered lipid status in manic patients remains mostly unclear. Cholesterol is a principal component of the cell membrane and plays an important role in synaptic functions through the organization of signaling components. The depletion of cholesterol may alter brain signaling, with effects on several neurotransmission systems. The aberration of serotoninergic function is among the most discussed mechanisms explaining the relationship between cholesterol and mood [50]. Cholesterol may influence this system through the postsynaptic serotonin receptor function and serotonin reuptake in the synapse. These alterations may be related to the onset of mood symptoms [51]. However, the involvement of other neurotransmission systems and physiological mediations suggest a complex relationship that has yet to be elucidated.

Other potential mechanisms underlying the decreased cholesterol levels in mania are related to the differences in nutritional status and motor activity. First, as for depression, appetite reduction may be linked to aberrant dietary patterns causing cholesterol depletion. Second, augmented motor activity may favor a decrease in the total and LDL cholesterol levels. It is worth noting that we did not detect any significant differences between manic and depressed patients in levels of HDL, the “good” cholesterol. This non-significant difference may also be linked to the poor nutritional status of both manic and depressed BD patients.

From a psychopathological point of view, low cholesterol levels have been traditionally associated with severe forms of MDD with suicide attempts [24,52,53], even if this issue is still under debate [22]. However, several studies have shown that decreased cholesterol levels are also typical of other symptom clusters, such as emotional lability, euphoria, impulsivity, irritability, and aggression, all clinical features of mania [50]. On the contrary, the association between the lipid profile and suicide attempts in BD has been disconfirmed by a recent meta-analysis [18]. It is relevant to the phenomenology of mania to determine if the decrease in cholesterol levels falls prior to or during manic episodes. On one hand, in fact, altered cholesterol levels may represent a signal of an incipient manic episode; on the other hand, they could be a consequence of aberrant lifestyle behaviors, diminished appetite with poor nutritional status, and hypermotricity. As cholesterol levels can be monitored through a simple and relatively low-cost blood examination, longitudinal studies with periodical clinical and laboratory follow-up should be implemented to further elucidate the relationship between low cholesterol levels and mood episodes—particularly, (hypo)mania—in BD.

### 4.2. Limitations

Despite the importance of our findings, several limitations should be acknowledged. First, the cross-sectional design did not allow to compare the glucose and lipid levels of the same individual while experiencing different phases of BD illness. In the future, we will plan to perform a longitudinal follow-up of patients with BD in order to evaluate changes in their lipid and glycemic profiles during different mood episodes. Second, the sample of patients with schizophrenia was smaller than the BD sample, and, given the “naturalistic” design of the study, it was not possible to match participants for sociodemographic variables and psychotropic medication use. Additionally, we had an imbalance between the number of BD participants in the euthymic phase (N = 60) and mood episodes (N = 122 for mania and N = 138 for depression). Third, some important parameters related to the metabolic profile and MetS (i.e., body mass index and blood pressure) were not included in the analyses due to the high number of missing values. That was also the case for the concomitant use of statins or antidiabetic medication, which may significantly influence the serum lipid and glucose levels, respectively. In future studies, we will consider these parameters in relation to the medical and psychiatric outcomes of patients with BD.

## 5. Conclusions

Patients with BD do not differ from patients with schizophrenia in the serum levels of glucose, cholesterol, or triglycerides, suggesting that psychotropic medication use and an unhealthy lifestyle may significantly impact the metabolic parameters in both groups. Thus, they should be periodically monitored since the first mood episode to prevent the onset of cardiovascular and metabolic complications. Interestingly, lower cholesterol levels in (hypo)manic patients compared to both euthymic and depressed patients evidenced a correlation between the metabolic profile and clinical symptoms. In the future, longitudinal well-designed studies should be conducted to better elucidate whether there is any causality link between the cholesterol levels and the onset of mood episodes in patients with BD. Indeed, the investigation of the interplay between the psychopathological, neurological, and biological variables may help to understand the complexity of several medical and psychiatric conditions, including BD [54].

## Figures and Tables

**Table 1:** Sociodemographic and clinical characteristics of the sample.

	**Total Sample** **(*N* = 542)**
Gender, *N* (%)	
Male	266 (49.1)
Female	276 (50.9)
Age (years), mean ± SD	52.62 ± 12.87
Marital status, single *N* (%)	295 (54.4)
Employment status, employed *N* (%)	180 (33.2)
Educational level (years), mean ± SD	11.51 ± 3.45
Primary diagnosis, *N* (%)	
Bipolar Disorder	418 (77.1)
Schizophrenia	124 (22.9)
Diagnosis of Bipolar Disorder, episode *N* (%)	
Major depressive episode	151 (26.4)
(Hypo)manic episode	143 (27.9)
Euthymia	63 (14.2)
Missing data	171 (31.5)
	**Pharmacological Treatment** (***N* = 479**)
N of psychiatric medications, mean ± SD	3.63 ± 1.18
Antidepressants, *N* (%)	173 (36.1)
Mood stabilizers, *N* (%)	
Valproate	179 (37.4)
Lithium	149 (31.1)
Others	98 (20.5)
Antipsychotics, *N* (%)	425 (88.7)
Typical	110 (23.0)
Atypical	372 (77.7)
Long-acting injectable	62 (12.9)
Benzodiazepines, *N* (%)	290 (60.5)
	**Metabolic Parameters** (***N* = 491**)
Total cholesterol, mean ± SD	185.23 ± 40.04
HDL cholesterol, mean ± SD	48.60 ± 13.97
LDL cholesterol, mean ± SD	110.70 ± 36.77
Triglycerides, mean ± SD	130.19 ± 71.78
Glucose, mean ± SD	96.52 ± 29.14

Legend: HDL: high-density lipoprotein, LDL: low-density lipoprotein, and SD: standard deviation.

**Table 2:** Sociodemographic, metabolic parameters, and medication prescriptions in patients with bipolar disorder and schizophrenia.

	**Bipolar Disorder (*N* = 418)**	**Schizophrenia** **(*N* = 124)**	**χ^2^/t**	***p***
Gender (male), *N* (%)	198 (47.4)	68 (54.8)	2.13	0.14
Age (years), mean ± SD	52.37 ± 13.35	53.44 ± 11.12	−0.81	0.42
Marital status, single *N* (%)	215 (51.4)	80 (64.5)	−4.53	**0.02**
Employment status, employed *N* (%)	150 (35.9)	30 (24.2)	2.89	0.08
Educational level (years), mean ± SD	11.83 ± 2.78	11.28 ± 4.18	0.62	0.32
Total cholesterol, mean ± SD (*N* = 444)	183.52 ± 38.96	190.31 ± 42.85	−1.63	0.10
HDL cholesterol, mean ± SD (*N* = 444)	48.92 ± 14.03	47.64 ± 13.77	0.88	0.38
LDL cholesterol, mean ± SD (*N* = 444)	108.91 ± 35.51	116.02 ± 39.96	−1.87	0.06
Triglycerides, mean ± SD (*N* = 444)	129.14 ± 71.02	133.27 ± 74.18	−0.55	0.58
Glucose, mean ± SD (*N* = 444)	96.40 ± 29.56	96.90 ± 27.85	−0.17	0.87
	**Bipolar Disorder** (***N*** = **358**)	**Schizophrenia** (***N*** = **121**)	**χ^2^/t**	***p***
N of psychiatric medications, mean ± SD	3.80 ± 1.18	3.11 ± 0.98	5.83	**<0.001**
Antidepressants, *N* (%)	153 (42.7)	20 (16.5)	26.92	**<0.001**
Mood stabilizers, *N* (%)				
Valproate	152 (42.5)	27 (22.3)	15.68	**<0.001**
Lithium	144 (40.2)	5 (4.1)	54.97	**<0.001**
Others	87 (24.3)	11 (9.1)	12.86	**<0.001**
Antipsychotics, *N* (%)	305 (85.2)	120 (99.2)	17.66	**<0.001**
Typical	62 (17.3)	48 (39.7)	25.54	**<0.001**
Atypical	279 (77.9)	93 (76.9)	0.06	0.806
Long-acting injectable	23 (6.4)	39 (32.2)	53.45	**<0.001**
Benzodiazepines, *N* (%)	238 (66.5)	52 (43.0)	20.92	<**0.001**

Legend: HDL: high-density lipoprotein, LDL: low-density lipoprotein, and SD: standard deviation.

**Table 3:** Comparison of metabolic parameters in patients with bipolar disorder (BD), according to their current mood episode.

Mean ± SD	Major Depressive Episode (*N* = 138)	(Hypo)manic Episode (*N* = 122)	Euthymic Phase (*N* = 60)	t	*p*	Tukey’s Post-Hoc		
						D vs. M	D vs. E	E vs. M
Total cholesterol	197.75 ± 39.87	167.66 ± 33.01	185.48 ± 39.90	21.02	**<0.001**	**<0.001**	N.S.	**0.008**
HDL cholesterol	49.54 ± 14.84	46.96 ± 12.91	52.18 ± 13.14	3.02	**0.05**	N.S.	N.S.	**0.04**
LDL cholesterol	121.10 ± 38.41	96.79 ± 29.47	110.01 ± 35.01	15.99	**<0.001**	**<0.001**	N.S.	**0.04**
Triglycerides	135.59 ± 71.79	119.56 ± 67.07	120.62 ± 66.59	2.03	0.13	N.S.	N.S.	N.S.
Glucose	97.04 ± 29.67	97.91 ± 32.29	98.05 ± 30.17	0.039	0.96	N.S.	N.S.	N.S.

Legend: HDL: high-density lipoprotein, LDL: low-density lipoprotein, SD: standard deviation, and N.S.: non-significant.

## Data Availability

The data presented in this study are available on request from the corresponding author. The data are not publicly available due to privacy/ethical restrictions.
